# Single cell transcriptome analyses reveal the roles of B cells in fructose-induced hypertension

**DOI:** 10.3389/fimmu.2023.1279439

**Published:** 2023-11-17

**Authors:** Cheong-Wun Kim, Sung Yong Joo, Boa Kim, Jee Young Kim, Sungmin Jang, Shiang-Jong Tzeng, Sang Jin Lee, Myunghoo Kim, Inkyeom Kim

**Affiliations:** ^1^ Department of Pharmacology, BK21 Plus Kyungpook National University (KNU) Biomedical Convergence Program, Cardiovascular Research Institute, School of Medicine, Kyungpook National University, Daegu, Republic of Korea; ^2^ Department of Animal Science, Pusan National University, Miryang, Republic of Korea; ^3^ Cardiovascular Research Institute, School of Medicine, Kyungpook National University, Daegu, Republic of Korea; ^4^ Graduate Institute of Pharmacology, College of Medicine, National Taiwan University, Taipei, Taiwan; ^5^ Division of Rheumatology, Cardiovascular Research Institute, School of Medicine, Kyungpook National University, Daegu, Republic of Korea

**Keywords:** single-cell RNA-sequencing, immunity, hypertension, B cell, interferon pathway

## Abstract

**Rationale:**

While the immune system plays a crucial role in the development of hypertension, the specific contributions of distinct immune cell populations remain incompletely understood. The emergence of single-cell RNA-sequencing (scRNA-seq) technology enables us to analyze the transcriptomes of individual immune cells and to assess the significance of each immune cell type in hypertension development.

**Objective:**

We aimed to investigate the hypothesis that B cells play a crucial role in the development of fructose-induced hypertension.

**Methods and Results:**

Eight-week-old Dahl salt-sensitive (SS) male rats were divided into two groups and given either tap water (TW) or a 20% fructose solution (HFS) for 4 weeks. Systolic blood pressure was measured using the tail-cuff method. ScRNA-seq analysis was performed on lamina propria cells (LPs) and peripheral blood mononuclear cells (PBMCs) obtained from SS rats subjected to either TW or HFS. The HFS treatment induced hypertension in the SS rats. The analysis revealed 27 clusters in LPs and 28 clusters in PBMCs, allowing for the identification and characterization of various immune cell types within each cluster. Specifically, B cells and follicular helper T (Tfh) cells were prominent in LPs, while B cells and M1 macrophages dominated PBMCs in the HFS group. Moreover, the HFS treatment triggered an increase in the number of B cells in both LPs and PBMCs, accompanied by activation of the interferon pathway.

**Conclusions:**

The significant involvement of B cells in intestinal and PBMC responses indicates their pivotal contribution to the development of hypertension. This finding suggests that targeting B cells could be a potential strategy to mitigate high blood pressure in fructose-induced hypertension. Moreover, the simultaneous increase in follicular B cells and Tfh cells in LPs, along with the upregulation of interferon pathway genes in B cells, underscores a potential autoimmune factor contributing to the pathogenesis of fructose-induced hypertension in the intestine.

## Graphical Abstract

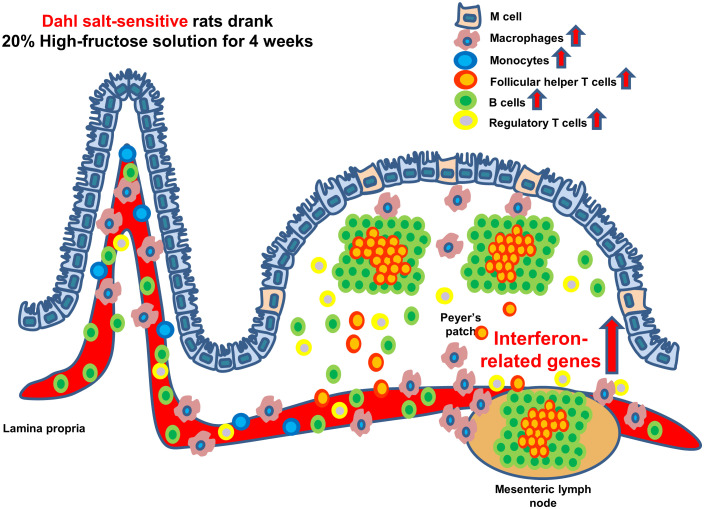

## 1 Introduction

The consumption of a high-fructose solution (HFS) is associated with an increase in blood pressure. Furthermore, combining a high-fructose with a high-salt diet induces salt-sensitive hypertension. These processes involve the activation of the renin-angiotensin-aldosterone system, changes in gut microbiota, increased sympathetic nervous system activity, enhanced reactive oxygen species (ROS), an increase in uric acid levels mediated by fructokinase, which leads to an upregulation of ion channels like Na^+^ -H^+^ exchanger 3 and Na^+^ -K^+^ -2Cl^-^ cotransporter (1).

The immune system plays a crucial role in the pathogenesis of hypertension (2, 3). The hallmarks of hypertension include the infiltration of innate and adaptive immune cells into perivascular fat, kidney, and myocardium, accompanied by elevated levels of cytokines, chemokines, adhesion molecules, and ROS. Studies using knock-in and knock-out mouse models have demonstrated the involvement of macrophages, monocytes, B cells, and T cells in hypertension (2). Moreover, excess fructose intake induces the secretion of pro-inflammatory cytokines, such as interleukin-6 (IL-6), tumor necrosis factor-α (TNF-α), and plasminogen activator inhibitor-1 (PAI-1) (4). Previous research has shown that administering a high-fructose diet induces the expression of serum/glucocorticoid-regulated kinase 1 (SGK1). In consequence, the induction of SGK1 expression leads to the phosphorylation of forkhead box O 1/3. As a result, this suppresses the expression of the master transcription factor forkhead box P3 (FoxP3) in regulatory T (Treg) cells and activates T helper 17 (Th17) cells to secrete IL23, further contributing to development of hypertension (5, 6).

The gastrointestinal (GI) tract, which is the largest mucosal tissue in both humans and animals, comprise epithelial, immunological, and vascular barriers and hosts a diverse community of gut microorganism (7, 8). The GI tract is continuously exposed to antigenic stimuli, and the epithelial layer and the lamina propria play crucial roles in immune responses. In particular, the lamina propria contains various immune cells, including cytotoxic T cells, B cells, Th cells, eosinophils, dendritic cells (DCs), and macrophages, which are key effector cells of the immune response. Furthermore, the intestinal immune cells are organized to form gut-associated lymphoid tissue, which comprise lymphoid structures such as Peyer’s patches, lymphoid follicles, and mesenteric lymph nodes (7, 9, 10). These organized structures regulate responses to both self and non-self-antigens. Dysregulated immune responses can lead to conditions such as food allergies and inflammatory bowel disease (7, 11).

Mounting evidence highlights the substantial involvement of the gut microbiome in the development and progression of hypertension, which is mediated by interactions with immune cells (12). Moreover, this link between the gut microbiome and hypertension is not limited to specific species (13). A study involving both mice and humans has found that a high-salt diet leads to a reduction in *Lactobacillus* spp. levels and an increase in blood pressure. Notably, the significance of using pro-biotic lactobacillus treatment should be emphasized, as it can suppress Th17 cells and ameliorate salt-sensitive hypertension (13, 14).

B cells recognize antigens and interact with Tfh cells, subsequently releasing cytokines that drive their differentiation into plasma cells responsible for antibody production (15). In murine models, the interaction between follicular helper T (Tfh) cells and germinal center (GC) B cells promotes the selection of B cells with the highest affinity for antigens through the expressions of CD40L, inducible T cell costimulatory (ICOS), and B cell activating factor (BAFF). However, B-cell immune responses extend beyond the GC environment. Extra-follicular B cell responses serve as early antibody sources during infection, maintaining elevated levels of G protein-coupled receptor 183 (GPR183) to avoid the GC milieu and increasing CXCR4 expression to facilitate their migration to lymph nodes (16–18). Dysregulation of GC and extra-follicular responses can result in the production of autoantibodies against self-antigens, contributing to the development of autoimmune diseases such as lupus and rheumatoid arthritis (17, 19).

While T cells have been well-recognized for their crucial role in the pathophysiology of hypertension, research into the connection between B cells and hypertension remains limited (20). Mice with simultaneous knockout of both T cells and B cells exhibit a blunted hypertensive response to angiotensin II (Ang II) stimulation. Interestingly, when wild type (WT) T cells and B cells are transplanted into the T cell-deficient and B cell-deficient mice, respectively, only the T cell transplantation exhibits a restoration of the hypertensive response to Ang II (21). However, depletion of B cells using anti-CD20 antibody and knocking out BAFF-receptor (BAFF-R) in mice result in a reduced increase in blood pressure induced by Ang II compared to WT mice. Furthermore, when WT B cells are transplanted into mice lacking BAFF-R, the hypertensive response is restored upon Ang II infusion (22).

In this study, we aimed to elucidate the immunological mechanisms underlying the development of hypertension induced by HFS in Dahl salt-sensitive (SS) rats. We performed single-cell RNA sequencing (scRNA-seq) on peripheral blood mononuclear cells (PBMCs) and lamina propria cells (LPs) isolated from SS rats receiving HFS (hereafter, “HFS group”). Our findings indicate an enlarged B cell population in the HFS group compared to the tap water (TW) group. Furthermore, our analysis using Gene Set Enrichment Assay (GSEA) and Differentially Expressed genes (DEGs) revealed a significant increase in interferon-related genes within B cells of the HFS group compared to the WT group.

## 2 Materials and methods

### 2.1 Animals

The *in vivo* experiments were conducted with the approval of the Kyungpook National University Institutional Review Board (Approval No. 2022-0456), following the guidelines outlined in the National Institutes of Health Guide for the Care and Use of Laboratory Animals. The study design aimed to minimize the number of animals used and to reduce the suffering of the experimental animals. Six-week-old Dahl-Iwai salt-sensitive (SS, DIS/EisSlc) male rats were purchased from Japan SLC, Inc (Hamamatsu, Shizoka, Japan). The rats had free access to a chow diet containing 0.4% NaCl (SAFE^®^ D 40, Paris, France) for one week to acclimate and were trained weekly for tail-cuff plethysmography. Subsequently, they were randomly assigned to either the high-fructose solution (HFS; 20% D-fructose; MB-F4695, Kisanbio, Korea) or tap water (TW) group for 4 weeks. The rats were anesthetized with sodium pentobarbital (50 mg/kg intraperitoneally) for euthanization, followed by the collection of tissues and PBMCs.

### 2.2 Blood pressure measurements

We measured the systolic blood pressure (SBP) of the SS rats using the tail-cuff method. The SS rats were placed on a hot plate (35°C) in a restraining device for 10 minutes. A cuff with a pneumatic pulse sensor was then attached to their tails. The CODA system (Kent Scientific Corporation, Torrington, CT, U.S.A.) was used to record blood pressure levels. To calculate the average blood pressure, at least five consecutive readings were obtained from each rat.

### 2.3 Isolation of LPs

LPs were isolated as described by Joo et al. (23). Briefly, jejunum tissues were cut into 0.5-cm pieces and washed with phosphate-buffered saline (PBS) containing 10 mM 4-[2-hydroxyethyl]-1-piperazineethanesulfonic acid (HEPES), 1 mM DL-dithiothreitol (DTT), and 30 mM ethylene-diamine-tetra acetic acid (EDTA; all from Thermo Fisher Scientific, Waltham, WA, U.S.A.) at 37°C for 10 minutes. Subsequently, the tissue samples underwent another wash in PBS containing 10 mM HEPES and 30 mM EDTA at 37°C for 10 minutes. After the wash, the tissues were transferred into 5 mL of RPMI 1640 (Gibco, Carlsbad, CA, U.S.A.) containing 10% fetal bovine serum (FBS) and inverted for 2 minutes. Following this step, the tissues were digested in RPMI 1640 containing 10% FBS with 0.5 mg/ml collagenase VIII (Sigma-Aldrich, St. Louis, MO, U.S.A.) at 37°C for 1 hour. After the digestion, isolated cells were applied to Percoll (GE Healthcare, Chicago, IL, U.S.A.) gradient centrifugation. LP samples were obtained after the removal of Peyer’s patches.

### 2.4 Isolation of PBMCs

Peripheral blood was collected from each rat, and PBMCs were isolated using Ficoll-Paque Plus^®^ gradient centrifugation (GE Healthcare, Chicago, IL, U.S.A.). The isolated PBMCs were washed with PBS and stored at room temperature for subsequent experiments.

### 2.5 Single-cell RNA library preparation and sequencing

The single-cell RNA library preparation and sequencing were performed by E-Biogen Inc. (Seoul, South Korea). The sequencing was conducted using the Nova-Seq 600 platform in a paired-end 100bp format, with 5000 cells sampled per sample. The library preparation method employed was the 10x Genomics Next Gem technology.

### 2.6 LPs and PBMCs clustering and annotation

We conducted the analysis using the filtered Cell Ranger files obtained from E-Biogen. The clustering of LPs and PBMCs was performed using the Seurat R Package (Seurat 4.4.0 version, https://satijalab.org/seurat/), a clustering tool developed for the merged matrix for scRNA-seq data. Potential doublets and low-quality cells (less than 200 genes or more than 10% of mitochondrial expression) were filtered out based on gene expression. The data were then normalized and combined. Variable genes were identified using the Seurat function “FindVariableGenes”. The previously identified variable genes were used for principal component analysis (PCA) to reduce the data dimensions. Subsequently, 30 principal components were utilized in the Uniform Manifold Approximation and Projection (UMAP) algorithm to further reduce the dimensions based on the neighborhood relationship. In the first round (pre-clustering; resolution = 0.8), major cell types were identified. In the second round (sub-clustering; resolution = 0.2), T cell or B cell subsets were further subdivided using specific signature genes. Gene expression was visualized using DotPlot, FeaturePlot, and VlnPlot functions from the Seurat package’s guidelines. Volcano plots were generated using the R package EnhancedVolcanoPlot.

### 2.7 Gene set enrichment assay

To explore the potential roles of B cells in both LPs and PBMCs, we utilized the Molecular Signature Database (MSigDB) and conducted GSEA. Data were analyzed using Seurat, dplyr, presto, msigdbr, fgsea, tibble, tidyverse, and data.table package in R. This analysis involved calculating a normalized enrichment score (NES), and was performed focusing on the most significant hallmark genes. GSEA results were visualized to identify functional or pathway differences. We used *Rattus norvegicus* as the species and categorized the results into the “H” category to specifically identify hallmark pathways in the HFS group.

### 2.8 Statistics

Blood pressure values were presented as the mean ± standard error of mean (SEM). Statistical analyses were performed using GraphPad Prism 7 (GraphPad Software, San Diego, CA, U.S.A.), and significance was determined with a *p* value of lower than 0.05. The Vlnplot of interferon-related genes was evaluated for significance using the Wilcoxon signed rank test.

## 3 Results

### 3.1 High-fructose intake increased blood pressure in SS rats

**Figure 1:**
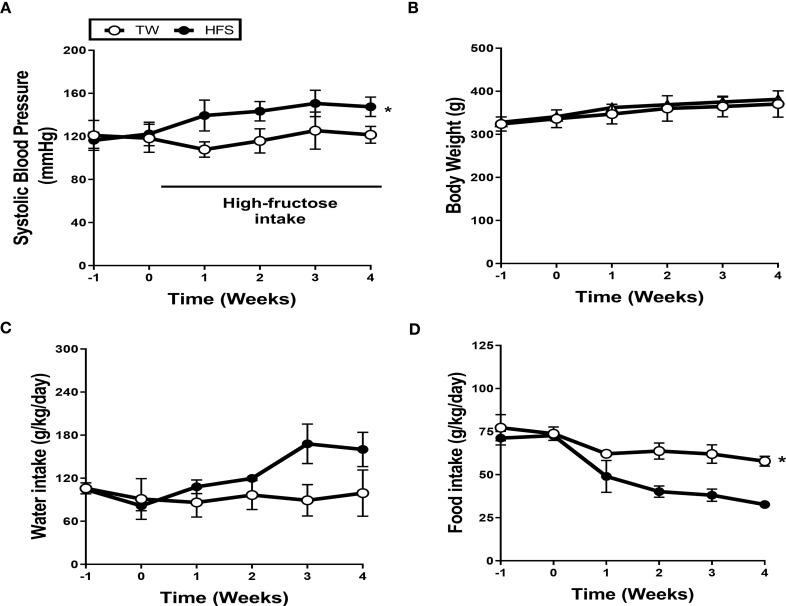
High-fructose intake induced hypertension. Dahl salt-sensitive (SS) rats were given either 20% high-fructose solution (HFS) or tap water (TW) for 4 weeks. **(A)** The HFS significantly increased systolic blood pressure (SBP). **(B, C)** There were no significant differences observed in body weight and water intake. **(D)** Conversely, the HFS group exhibited a notable reduction in food intake. The graph represents the mean ± SEM of eight independent experiments. The statistical analysis involved conducting a repeated measures ANOVA followed by Tukey’s *post-hoc* multiple comparisons test. The asterisk (*) indicates statistical significance (*p* < 0.05) compared to the TW group. The study was conducted with a sample size of n = 4.

To determine the effect of a high-fructose solution on blood pressure, we conducted weekly measurements of systolic blood pressure (SBP) in SS rats over a 4-week period. The rats were divided into two groups: one group receiving tap water (TW) and the other receiving a 20% high-fructose solution (HFS). The results showed a significant increase in SBP and mean blood pressure in the HFS group for 4 weeks, but no significant change was observed in the TW group (**Figures 1A** and **S1B**). There were no significant differences in diastolic blood pressure, heart rate, calorie intake, body weight, and water intake between the groups (**Figures 1B, C**, **S1A, S1C, S2**). However, the HFS group exhibited a decrease in food intake (**Figure 1D**).

### 3.2 Landscape of major cell types in LPs of TW and HFS group

**Figure 2:**
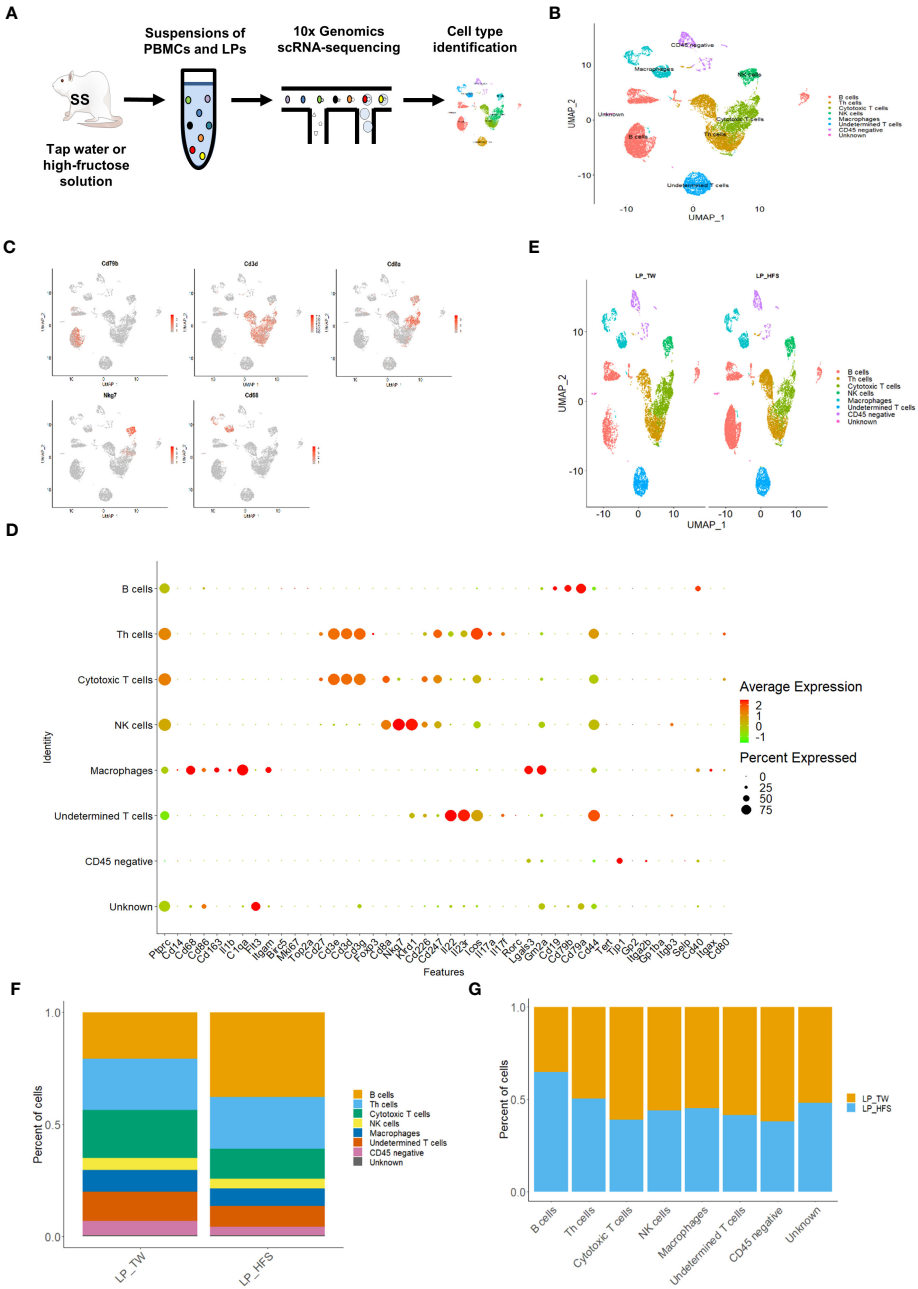
High-fructose intake increased B cells within the lamina propria cells (LPs). **(A)** Schematic representation of the experimental strategy. **(B)** The UMAP plot of LPs revealed five major immune cell types, along with CD45 (protein tyrosine phosphatase receptor type C; *Ptprc*)-negative cells, undetermined T cells, and unknown cell types. **(C)** The feature plots demonstrate pronounced expression of *CD68* in macrophages, *Nkg7* in NK cells, *Cd3d* in Th cells, *Cd8a* in cytotoxic T cells, and *Cd79b* in B cells. **(D)** The dot plot of selected marker genes for each cell type. The size of dots represents the percentage of gene expression in each cell subset, while the color of dots indicates the expression levels. **(E)** The HFS group showed an increase in B cells (in red) compared to the TW group. The UMAP plots compare the distribution of the cell types across the TW and HFS groups. **(F, G)** The proportion of B cells was notably higher in the HFS group than in the TW group. **(F)** Depiction of the cell type proportions in each group. **(G)** The relative quantification of each cell type between the two groups.

We performed scRNA-seq on PBMCs (n = 3) and LPs (n = 4) obtained from the TW and HFS groups (**Figure 2A**). The datasets from these groups were combined for further analysis. After quality control, we successfully processed an average of 3445 cells and 3481 cells in the LPs of the TW and HFS groups, respectively. The major cell types were classified and identified as B cells, helper T (Th) cells, cytotoxic T cells, natural killer (NK) cells, and macrophages. These cell types were visualized using a UMAP plot (**Figure 2B**), with their identification based on specific marker genes. B cells were characterized by the marker genes *Cd19*, *Cd79a*, and *Cd79b*, while Th cells were distinguished by *Cd3d*, *Cd3e*, and *Cd3g*. Cytotoxic T cells were marked by *Cd3d*, *Cd3g*, and *Cd8a*, NK cells by *Cd8a*, *Nkg7*, and *Klrd1*, and macrophages by *Cd14*, *Cd68*, *Cd86*, and *Cd163*. These cell types and their corresponding marker genes were visualized through dot plots and feature plots (**Figures 2C, D**). A comparison of UMAP plots between the groups highlighted a greater abundance of B cell clusters in the HFS group compared to the TW group (**Figure 2E**). This observation was further corroborated through a bar graph, illustrating the increased proportion of B cells in the HFS group compared to the TW group (**Figures 2F, G**).

### 3.3 Increased follicular helper T cells in the T cell subsets of LPs from the HFS group

**Figure 3:**
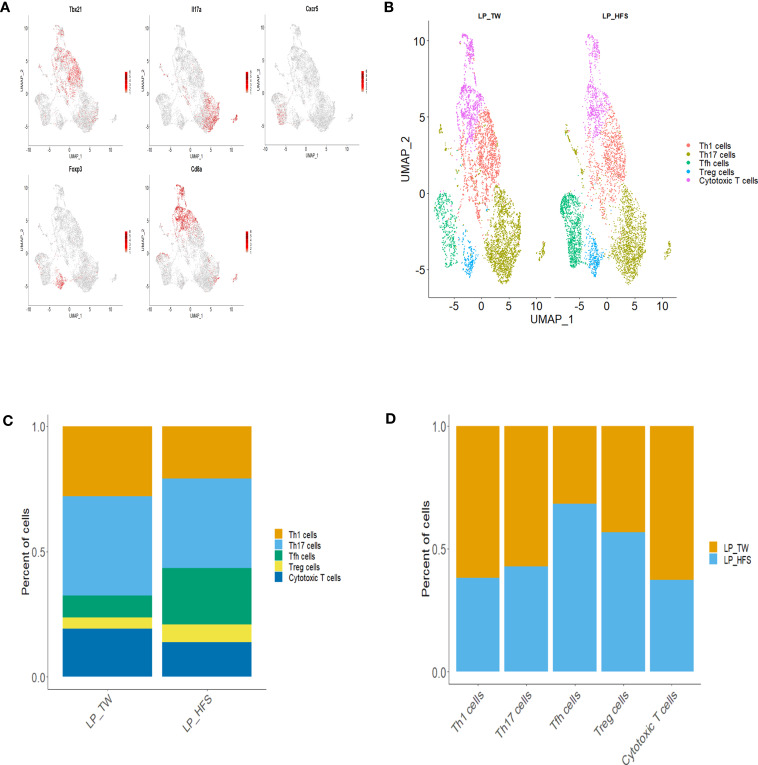
High-fructose intake increased the follicular helper T cell subset in LPs. **(A, B)** The HFS group displayed an increased presence of Tfh cells (in green) compared to the TW group. **(A)** The feature plots demonstrate high expression of *Tbx21* in Th1 cells, *Cxcr5* in Tfh cells, *Il17a* in Th17 cells, *Foxp3* in Treg cells, and *Cd8a* in cytotoxic T cells. **(C, D)** The proportion of Tfh and regulatory T (Treg) cells was higher in the HFS group compared to the TW group. **(B)** The UMAP plot enables a visual comparison of the distribution of T cell subsets between the TW and HFS groups. **(C)** Depiction of the proportions of T cell subsets in each group. **(D)** The relative amount of each T cell subset between the two groups.

To explore the diversity of T cell subsets within the major LP clusters, we conducted sub-clustering with a focus on the Th cell and cytotoxic T cell clusters. As a result, T cell subsets, including Th1, Th17, Tfh, Treg, and cytotoxic T cells, were identified. The identification of T cell subsets was based on specific marker genes. Th1 cells were identified by *Ccr5*, *Cxcr3*, *Stat4*, and *Tbx21*. Th17 cells were defined by *Ccr6*, *Il23r*, *Il17a*, and *Il17f*. Tfh cells were delineated by *Cxcr5*, *Il6r*, and *Stat3*. Treg cells were categorized using *Il2ra*, *Ctla4*, and *Foxp3*. Cytotoxic T cells were pinpointed through *Cd8a*, *Gzmm*, and *Nkg7*. These T cell subsets, along with their characteristic marker genes, were visually presented through a dot plot and feature plots (**Figures 3A** and **S3**). Visualization of these T cell subsets were achieved through UMAP plots, and a comparison between the two groups revealed a higher presence of Tfh cells in the HFS group (**Figure 3B**). This observation was further supported by the bar graph, which displayed a higher proportion of Tfh cells within LPs from the HFS group compared to the TW group (**Figures 3C, D**).

### 3.4 Elevated levels of follicular B cells in the B cell subsets of LPs from the HFS group

**Figure 4:**
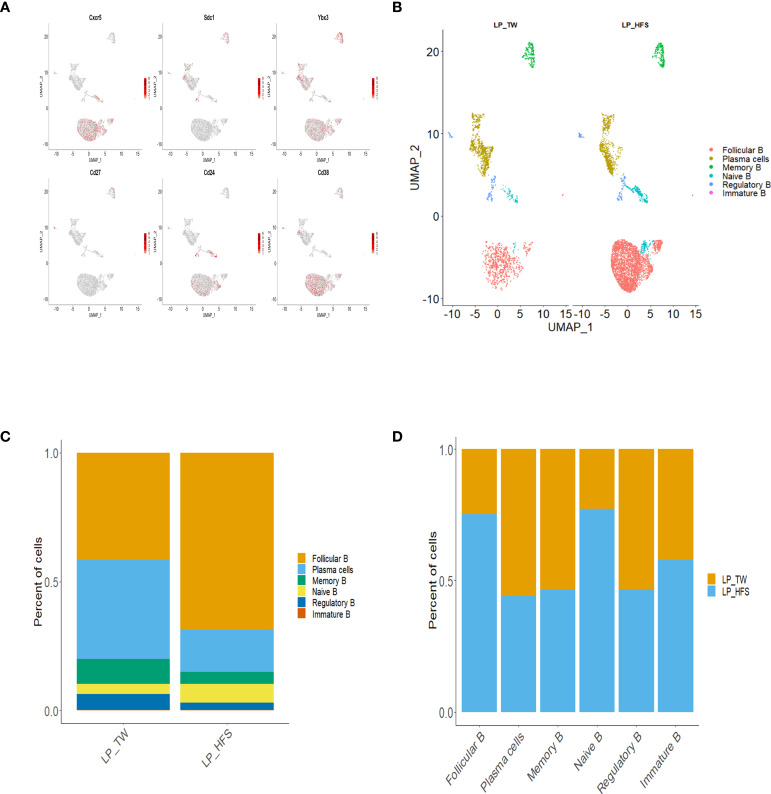
High-fructose intake increased the follicular B cell subset in LPs. **(A, B)** The HFS group exhibited an increase in follicular B cells compared to the TW group. **(A)** The feature plots demonstrate high expression of *Cxcr5* and *Cd38* in follicular B cells, *Sdc1* in plasma cells, *Ybx3* in immature B cells, *Cd27* in memory B cells, *Cd24* and *Sdc1* in regulatory B cells, and *Cd24* in naïve B cells, with low expression of *Cd38*. **(B)** The UMAP plot compares the distribution of B cell subsets across the TW and HFS groups. **(C, D)** The proportion of follicular B cells was higher in the HFS group than in the TW group. **(C)** The proportion of B cell subsets in each group. **(D)** The relative amount of each B cell subset between the two groups.

Our exploration of B cell diversity within the major LP clusters involved sub-clustering, focusing particularly on the B cell clusters. By referencing established works (24–26), we conducted the annotation of B cell subsets and identified distinct B cell subsets: follicular B, memory B, Naïve B, regulatory B (Breg), immature B, and plasma cells. The B cell subsets were further visualized using UMAP plots. The characterization of B cell subsets was based on specific marker genes. Follicular B cells were classified by their high expression of *Cxcr5, Cd24*, and *Cd38*. Memory B cells were identified based on their high expression of *Cd27* and *Cd38*. Naïve B cells were recognized by their high expression of *Cxcr5* and *Cd24*, accompanied by low expression of *Cd38*. Breg cells were identified through their high expression of *Cd24* and *Cd38*. Immature B cells were identified by their high expression of *Ybx3* and *Cd38*. Plasma cells were categorized by their high expression of *Sdc1* and *Ighm*, coupled with low expression of *Ptprc* and *Cd24.* These B cell subsets, along with their associated marker genes, were visually presented through a dot plot and feature plots (**Figures 4A** and **S4**). A comparative analysis of these B cell subsets between the two groups revealed a higher abundance of follicular B cells in the HFS group (**Figure 4B**). This trend was supported by a bar graph, which showed a higher proportion of follicular B cells in LPs from the HFS group compared to the TW group (**Figures 4C, D**).

### 3.5 Landscape of major cell types from PBMCs of TW and HFS group

**Figure 5:**
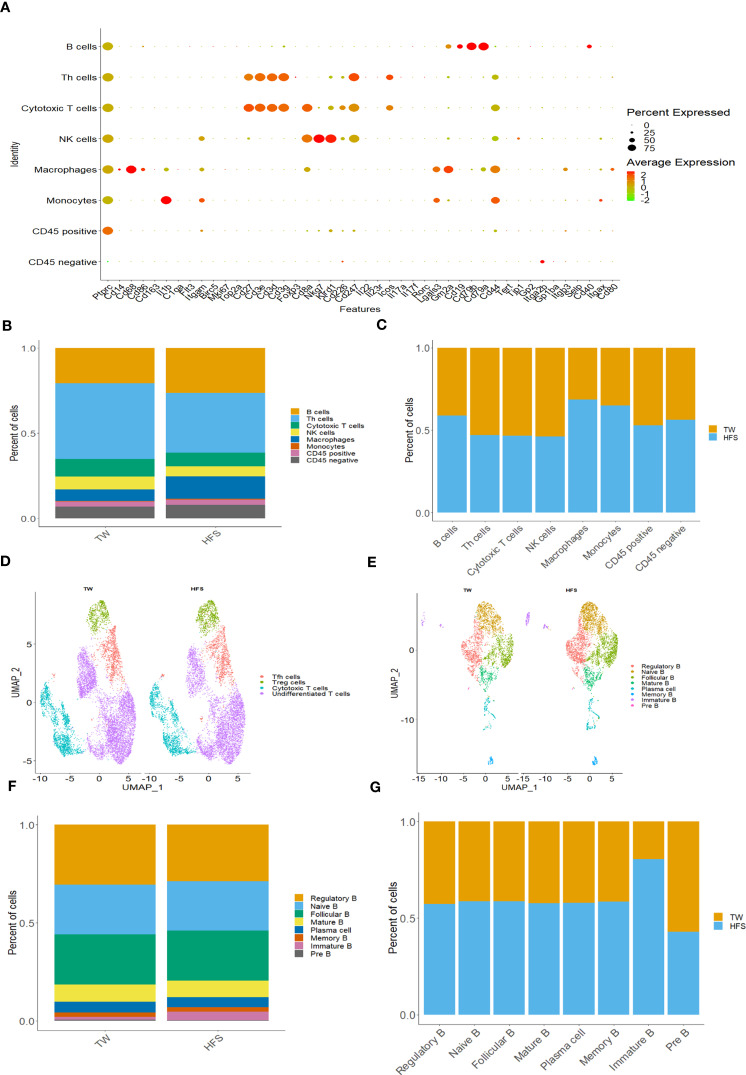
High-fructose intake increased B cells, monocytes, and macrophages within peripheral blood mononuclear cells (PBMCs). **(A-C)** The HFS group exhibited an increase in B cells, macrophages, and monocytes compared to the TW group. **(A)** The dot plot of selected marker genes for each cell type. Dot size indicates the percentage of each gene expressed in each cell type, while dot color indicates expression levels. **(B)** The proportion of cell types in each group. **(C)** The relative amount of each cell type between the two groups. **(D)** The UMAP plot compares the distribution of the T cell subsets across the TW and HFS groups. **(E-G)** HFS increased almost all B cell subsets in HFS group compared to TW group in PBMCs. **(E)** The UMAP plot compares the B cell subsets distribution across TW and HFS groups. **(F)** The proportion of B cell subsets in each group. **(G)** The relative amount of each B cell subtype between the two groups.

We analyzed scRNA-seq datasets from PBMCs of the TW and HFS groups. Subsequently, these datasets were merged for comprehensive scrutiny. Following rigorous quality control measures, an average of 5255 cells in the TW group and 5886 cells in the HFS group were successfully acquired within the PBMCs. Among these, major cell types were identified and classified as B cells, Th cells, cytotoxic T cells, NK cells, macrophages, and monocytes. B cells were identified using *Cd19*, *Cd79a*, and *Cd79b*. Th cells were confirmed using *Cd3d*, *Cd3e*, and *Cd3g*. Cytotoxic T cells were validated through *Cd3d*, *Cd3g*, and *Cd8a*. NK cells were pinpointed using *Cd8a*, *Nkg7*, and *Klrd1*. Macrophages and monocytes were precisely identified using *Cd14*, *Cd68*, *Cd86*, and *Lgals3*, *Gm2a*, *Itgam*, respectively. This comprehensive categorization was visually presented in a dot plot (**Figure 5A**). These cell types were visualized using bar plots (**Figures 5B, C**), and their identification was based on specific marker genes.

To further explore T cell and B cell subsets, we visualized them through UMAP plots. The classification of T cell subsets included undifferentiated T cells, cytotoxic T cells, Tfh cells, and Treg cells. These distinctions were made based on specific marker genes. Cytotoxic T cells were defined by *Cd8a*, *Cd8b*, *Gzmm*, and *Nkg7*. Tfh cells were identified using *Cxcr5*, *Icos*, and *Il6r*. Treg cells were identified using *Il2ra*, *Ctla4*, and *Foxp3*. The results of this comprehensive categorization were summarized in a dot plot (**Figure S5A**). Similarly, B cell subsets exhibited a wide range of diversity, including plasma cells, Breg cells, naïve B cells, follicular B cells, mature B cells, memory B cells, immature B cells, and pre B cells. The identification of these subsets was based on distinct marker genes. Breg cells were identified by their high expression of *Cd19*, *Cd24*, *Ms4a1*, and *Cd38*. Mature B cells were classified by their high expression of *Cd19* and *Pax5*, with low expression of *CD79a.* Follicular B cells exhibited high expression of *Cxcr5* and *Cd19*, coupled with low expression of *Cd27*. Immature B cells were recognized by their high expression of *Ybx3* and *Cd38*. Memory B cells were classified based on high expression of *Cd27* and *Cd38*. Plasma cells were distinguished by their high expression of *Sdc1* and *Ighm*, alongside low expression of *Ptprc* and *Cd24.* Pre B cells were identified based on their high expression of *Cd19* and low expression of *Ms4a1*. The diversity of B cells was illustrated in a dot plot (**Figure S6**). Further investigation of UMAP plots and bar plots between the two groups in PBMCs revealed a noteworthy trend: within T cell subsets, the HFS group exhibited a higher abundance of Treg cells, while nearly all B cells in the HFS group displayed a greater prevalence (**Figures 5D-G** and **S5B, S5C**).

### 3.6 Activation of the interferon signaling pathway in B cells of PBMCs and LPs from the HFS group

**Figure 6:**
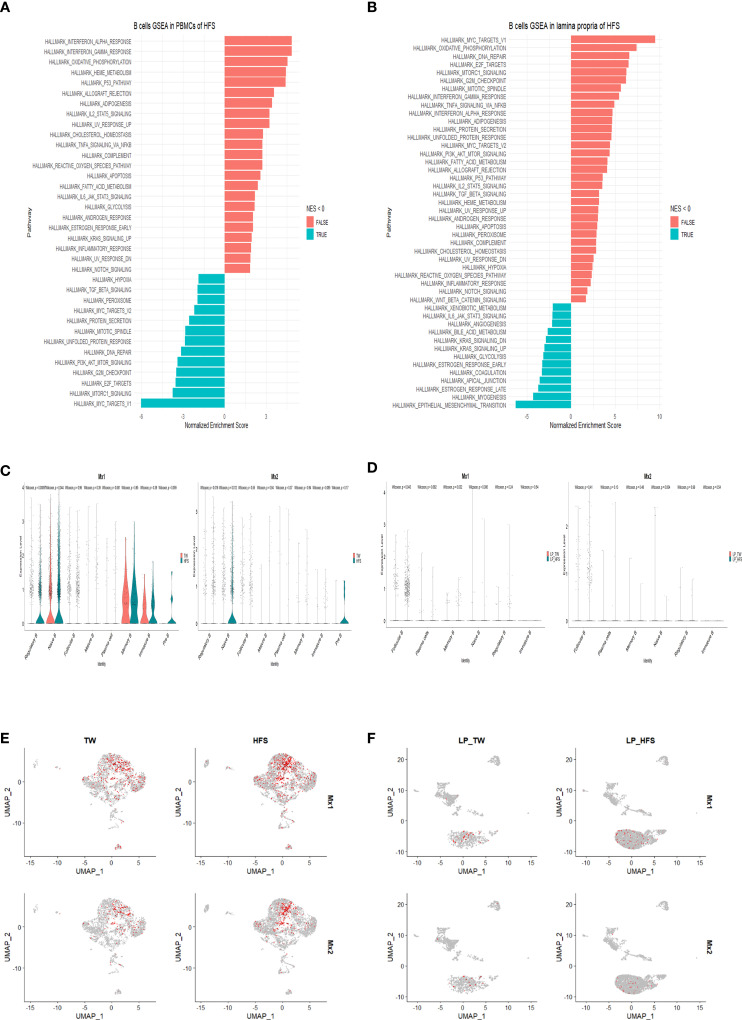
High-fructose intake upregulated the expression of interferon-related genes in B cells found in PBMCs and LPs. **(A, B)** The hallmark pathways, including “INTERFERON_ALPHA_RESPONSE” and “INTERFERON_GAMMA_RESPONSE”, were upregulated in B cells from the HFS group. **(A)** Utilizing gene set enrichment analysis (GSEA) and normalized enrichment score (NES), enriched hallmark gene sets in B cells from PBMCs of the HFS group were identified compared to the TW group. **(B)** Similarly, GSEA and NES were applied to identify enriched hallmark gene sets in B cells from LPs from the HFS group compared to the TW group. Gene sets with a NES < 0 are shown as leftward bars (in blue), whereas gene sets with NES > 0 are shown as rightward bars (in red). **(C-F)** Notably, the expression of ISGs was increased in B cell subsets of both PBMCs and LPs from the HFS group compared to the TW group. **(C)** The violin plots display the ISG expression, including *myxovirus resistance-1* (*Mx1*) and *myxovirus resistance-2* (*Mx2*), within each B cell subset of PBMCs. The TW group is depicted in red and the HFS group in blue on the vln plots. **(D)** The violin plots display the expression of ISGs such as *Mx1* and *Mx2* within each B cell subset of LPs. The TW group is depicted in red and the HFS group in blue on the vln plots. **(E)** The feature plots illustrate the expression of ISGs such as *Mx1* and *Mx2* within each B cell subset of PBMCs. **(F)** The feature plots illustrate the expression of ISGs such as *Mx1* and *Mx2* within each B cell subset of LPs.

To elucidate the differences between the TW and HFS groups, we conducted gene set enrichment analysis (GSEA) on B cells. The GSEA results unveiled the upregulation of hallmark pathways “INTERFERON_ALPHA_RESPONSE” and “INTERFERON_GAMMA_RESPONSE” in B cells from the HFS group (**Figures 6A, B**). In addition, Differentially Expressed Genes (DEGs) analysis was performed on T cells and B cells to investigate the gene expression differences between the TW and HFS groups. The enhanced volcano plots unveiled elevated expression of ISGs, in the enhanced volcano plots of T cells from both the TW and HFS groups, genes associated with Treg cells and ISGs, such as *Foxp3* and *Ifitm1*, exhibited higher expression in PBMCs. Furthermore, in B cells, such as *Ifi30*, *Mx1*, and *Mx2* were found to be increased (**Figures S7A**, **S7B**). Moreover, genes related to Treg and Tfh cells that were found to be increased in LPs included *Icos* and *Foxp3*, and genes such as *Ifitm1* and *Ifitm3* in B cells were also increased in the HFS group compared to the TW group (**Figures S7C**, **S7D**). Comparative analysis of B cell clusters from PBMCs and LPs using violin (vln) plots reveled distinct expression of IFN-stimulated genes (ISGs). Specifically, in PBMCs, *Mx1* showed significantly elevated expression in Breg, while *Mx2* was significantly upregulated in Breg and naïve B in the HFS group. In LPs, *Mx1* showed significantly elevated expression in follicular B cells in the HFS group (**Figures 6C, D**). These observations were further supported by the feature plots, which depicted an increased proportion of ISGs in B cells of both PBMCs and LPs from the HFS group (**Figures 6E, F**). When comparing the expression levels of other interferon-related genes using vln plots in T cell subsets of PBMCs, we observed elevated *Ifnar2 and Ifngr1* were significantly upregulated in Tfh cells of PBMCs from the HFS group (**Figure S8**). In B cells, *Ifi30* and *Ifngr1* were significantly upregulated in plasma cells, and *Ifngr1* showed significantly elevated expression in follicular B cells of PBMCs from the HFS group compared to the TW group (**Figure S9**). Moreover, further analysis of the interferon-related genes in the T cell subsets of LPs revealed that *Irf1, Ifi30, Ifitm1, Ifnar1, and Ifngr2* were significantly upregulated in Tfh cells in the HFS group (**Figure S10**). In B cells, *Irf1, Stat2, Ifi30, Ifngr1, Ifngr2*, *Ifnar1* and *Ifnar2* were significantly upregulated in follicular B cells of LPs from the HFS group compared to the TW group (**Figure S11**). Furthermore, a significant increase in *IFN-γ* was observed in Th1 cells from LPs with HFS (**Figure S12**). These results collectively suggest a comprehensive activation of interferon-related genes in B cells from PBMCs and LPs in the context of hypertension induced by HFS.

## 4 Discussion

In this study, we provide evidence that HFS leads to an increase in blood pressure, linked to the activation of the IFN signaling pathway in B cells within both LPs and PBMCs. Notably, we observed that the administration of HFS led to an augmented population of B cells among the major cell types in both LPs and PBMCs. Moreover, our investigation uncovered distinct subsets within T cells and B cells: Tfh and Treg cells exhibited an increase within T cell subsets, while follicular B cells showed an elevation in B cell subsets from LPs in the HFS group. Moreover, Treg cells demonstrated an increase in T cell subsets, and B cells (excluding pre B cells) were elevated in B cell subsets from PBMCs in the HFS group.

Our findings indicate that HFS increases SBP through the activation of both the type I and type II IFN signaling pathways in B cells derived from both LPs and PBMCs. The IFN family consists of two main classes: type I IFNs (IFN-α, β, δ, ϵ, κ, τ, and ω), and type II IFN (IFN-γ) (27, 28). Type I IFNs are produced by various cell types. For instance, IFN-α is produced by plasmacytoid DCs, while IFN-β originates from fibroblasts, epithelial cells, macrophages, and monocytes (29, 30). Recent studies have reported that B cells can autonomously produce IFN-α and IFN-β (31, 32). Interestingly, type I IFN has been associated with pulmonary arterial hypertension (PAH) and autoimmune diseases in both humans and mice (33, 34). On the other hand, the predominant producers of type II IFN are Th1 cells, cytotoxic T cells, and NK cells (35, 36), and this cytokine has been associated with the development of hypertension. Intriguingly, the absence of IFN-γ has been shown to result in a blunted increase in SBP in animal models of hypertension induced by Ang II or deoxycorticosterone acetate (DOCA) in combination with salt (37, 38). Previous studies have demonstrated that B cells also secrete both types of IFNs as well as various cytokines (36, 39, 40). Furthermore, both type I and type II IFNs induce the expression of ISGs, which possess diverse immunomodulatory activities and are associated with hypertension (41). Among the ISGs, *Ifi30*, derived from DEGs of monocytes and macrophages, has been correlated with hypertension in patients with abdominal aortic aneurism or intracranial aneurism (42). The upregulation of the *Mx1* and *Mx2* genes has also been linked to inherited stress-induced arterial hypertension in rats (43).

We have observed an increase in Tfh cells in the LPs of the HFS group. These Tfh cells play a crucial role in assisting B cells, particularly follicular B cells, within germinal center (GC) reactions. GC reactions are important for effective immune responses against invading pathogens, but the dysregulation of Tfh cells has been implicated in various autoimmune diseases, inflammatory conditions, and B cell-related malignancies (19). Furthermore, Tfh cells have been linked to the development of hypertension (44, 45). Tfh cells are recognized for their secretion of IL-21, which fosters the activation of GC B cells within secondary or tertiary lymphoid organs. The IL-21 derived from Tfh cells plays a critical role in processes such as immunoglobulin (Ig) class switching and the generation of high-affinity antibodies by GC B cells (46, 47). Notably, when Ang II is administered into IL-21 knockout mice versus WT mice, IL-21 deficiency is associated with lower blood pressure, diminished vascular and end-organ damage, and reduced levels of IL-17A and IFN-γ compared to WT mice. Moreover, the neutralization of IL-21 results in decreased blood pressure, alleviation of vascular inflammation, and improvement in endothelial dysfunction. Importantly, human subjects with hypertension exhibit higher levels of IL-21 in their PBMCs compared to individuals with normal blood pressure (46).

Our research demonstrates that the consumption of HFS leads to an increase in B cells in both LPs and PBMCs. Recent evidence underscores the potential contribution of B cells in the development and progression of hypertension and vascular injury. The infusion of Ang II has revealed a surge in activated B cells and plasma cells. Moreover, studies using mice lacking the B cell activating factor receptor (BAFF-R) have shown prevention of blood pressure elevation and a reduction in aortic macrophage infiltration. Remarkably, the adoptive transfer of B cells from WT mice into those lacking BAFF-R reinstates their susceptibility to hypertension. Notably, the depletion of B cells by anti-CD20 antibodies in combination with Ang II administration results in a significant decrease in blood pressure (22). Furthermore, mice deficient in the B cell transcription factor c-myb, which is essential for the development of mature B cells, exhibit lower blood pressure compared to their WT littermates (48).

Hence, when implementing a high-fructose diet, blood pressure elevates through non-immune mechanisms. Subsequently, the activation of macrophages and DC cells is induced by specific antigens associated with hypertension, including neo-antigens and damage associated molecular patterns (DAMPs). These activated immune cells then proceed to stimulate T cells in the spleen, which, in turn, activate B cells to produce auto-antibodies. Consequently, this process leads to more extensive damage to various tissues such as blood vessels, kidneys, and the heart, ultimately progressing into more severe hypertension (49). Furthermore, a high-fructose diet may induce changes in gut microbiota composition, potentially activating B cells. For example, in a systemic sclerosis mouse model, when a high-fructose diet was administered for 4 and 12 weeks, *Bifidobacterium pseudomonas* and *Muribacterium intestinale* species in the gut microbiota decreased while *Olsenella timonensis* and *Desulfovibrio vulgaris* species increased. Subsequently, we observed a trend of increased B cells and Treg cells in the ileum 4 weeks after the high-fructose diet (50). Another example can be seen in non-alcoholic steatohepatitis (NASH), where a 20-week high-fructose diet resulted in the significant accumulation of pro-inflammatory B cells in the liver. This was attributed to factors induced by changes in gut microbiota (51). Consequently, we speculate, based on our experimental findings, that alterations in gut microbiota composition due to a high-fructose diet may activate B cells, potentially impacting not only systemic sclerosis and non-alcoholic fatty liver disease (NAFLD), but also hypertension.

Our study reveals a notable increase in the Treg population in both LPs and PBMCs from the HFS group. Treg cells not only play a crucial role as key regulators of inflammation but also have a dual role in maintaining self-tolerance and protecting against autoimmune diseases. The stability and function of the Treg cell lineage rely on the signaling mediated by the transcription factor Foxp3 (52, 53). These cells employ diverse mechanisms to suppress antigen presentation, including the induction of IL-10 expression as well as the inhibition of DC maturation (54, 55). Moreover, Treg cells control T cell proliferation and differentiation through upregulation of the expression of granzyme B and CD73, while simultaneously dampening the production of multiple cytokines such as IL-2, IFN-γ, and TNF-α (56). Previous investigations have reported the role of Treg cells in inhibiting B cell activity, both *in vitro* and *in vivo*, through mechanisms that depend on cellular contact (57, 58). Other studies have suggested that B cells are essential for the proliferation and expansion of not only antigen-primed effector Th cells but also Treg cells (40, 59, 60). Based on our findings, it is reasonable to propose that the observed augmentation in Treg cells could serve as a compensatory response aimed at counterbalancing the effects of B cells in LPs and PBMCs. The elevated expression of IFNs and ISGs within these B cells implies a connection to immune regulatory processes, potentially associated with autoimmunity. This aligns with recent evidence highlighting the connection between inflammation, autoimmunity, and the development of hypertension (34).

In our previous findings, the application of an HFS induced an imbalance in SGK1-Foxo1/3 signaling. Th17 cells increased in PBMCs and the spleen of Dahl salt-sensitive rats, while the Treg cells remained unaffected (6). However, upon verification through scRNA-Seq data, we observed that in the LP, there was no significant difference in the expression of SGK1 and Foxo1/3 when a HFS was applied. Th17 cells exhibited a tendency to decrease as compared to the WT. Although we were unable to pinpoint the exact causes, it is speculated that Th17 cells play a minimal role in the LP when hypertension occurs. This is likely due to tissue and environmental characteristics. For example, in previous studies, it has been well-documented that in rodent models, when CD8^+^ T cells are transferred or high-salt diets are administered, CD8^+^ T cells become established in the kidneys, contributing to the induction of hypertension (61). Moreover, when mice with knockout of IFN-γ, a cytokine secreted by CD8^+^ T cells, were subjected to DOCA-salt treatment, they showed lower blood pressure as compared to WT mice. Additionally, there was a reduced presence of CD8^+^ T cells in the kidneys (38). This evidence strongly supports the association of CD8^+^ T cells with the development of hypertension in the kidneys. However, a study examining the relationship between hypertension history and the gut in humans revealed a reduction in CD8^+^ T cells in the intestines of individuals with hypertension as compared to those with healthy intestines (62).

In summary, our study demonstrates that high fructose intake leads to an increase in blood pressure. This increase is driven by the activation of the IFN signaling pathway in B cells in both LPs and PBMCs. These findings suggest that targeting B cells could be a potential intervention strategy to reduce blood pressure in individuals with fructose-induced hypertension.

## Data availability statement

The data presented in the study are deposited in the NCBI Sequencing Read Archive database, accession number PRJNA1013706, at the following link: http://www.ncbi.nlm.nih.gov/sra/?term=PRJNA1013706.

## Ethics statement

The animal study was approved by Kyungpook National University Institutional Review Board. The study was conducted in accordance with the local legislation and institutional requirements.

## Author contributions

C-WK: Data curation, Formal Analysis, Investigation, Methodology, Validation, Visualization, Writing – original draft. SJ: Investigation, Methodology, Writing – review & editing. BK: Investigation, Writing – review & editing. JK: Investigation, Writing – review & editing. SJ: Investigation, Writing – review & editing. S-JT: Writing – review & editing. SL: Writing – review & editing, Data curation. MK: Writing – review & editing, Data curation. IK: Conceptualization, Funding acquisition, Project administration, Supervision, Writing – review & editing, Data curation.
