# Supplementary material for: Single cell transcriptome analyses reveal the roles of B cells in fructose-induced hypertension
Source: Front Immunol. 2023 Nov 17;14:1279439. doi: 10.3389/fimmu.2023.1279439 (PMC10691591; doi:10.3389/fimmu.2023.1279439)
Supplement: Supplementary file 1 [file DataSheet_1.docx]

Supplementary Material

**Single cell transcriptome analyses reveal the roles of B cells in fructose-induced hypertension**

Cheong-Wun Kim^1^, Sung Yong Joo^2^, Boa Kim^3^, Jee Young Kim^1^, Sungmin Jang^1^, Shiang-Jong Tzeng^4^, Sang Jin Lee^5^, Myunghoo Kim^2^, and InKyeom Kim^1^

^1^Department of Pharmacology, BK21 Plus KNU Biomedical Convergence Program, Cardiovascular Research Institute, School of Medicine, Kyungpook National University, 41944, Republic of Korea; ^2^Department of Animal Science, Pusan National University, 50463, Republic of Korea; ^3^Cardiovascular Research Institute, School of Medicine, Kyungpook National University, 41944, Republic of Korea; ^4^Graduate Institute of Pharmacology, College of Medicine, National Taiwan University, Taipei 10051, Taiwan; ^5^Division of Reumatology, Cardiovascular Research Institute, School of Medicine, Kyungpook National University, 41944, Republic of Korea

***Corresponding author:** Shiang-Jong Tzeng, Sang Jin Lee, Myunghoo Kim, and Inkyeom Kim

Tel: +82-10-6546-6933 Fax: +82-50-4009-6933

ORCID: http://orcid.org/0000-0001-8009-5801

E-mail: [inkim@knu.ac.kr](mailto:inkim@knu.ac.kr)

## Supplementary Figures


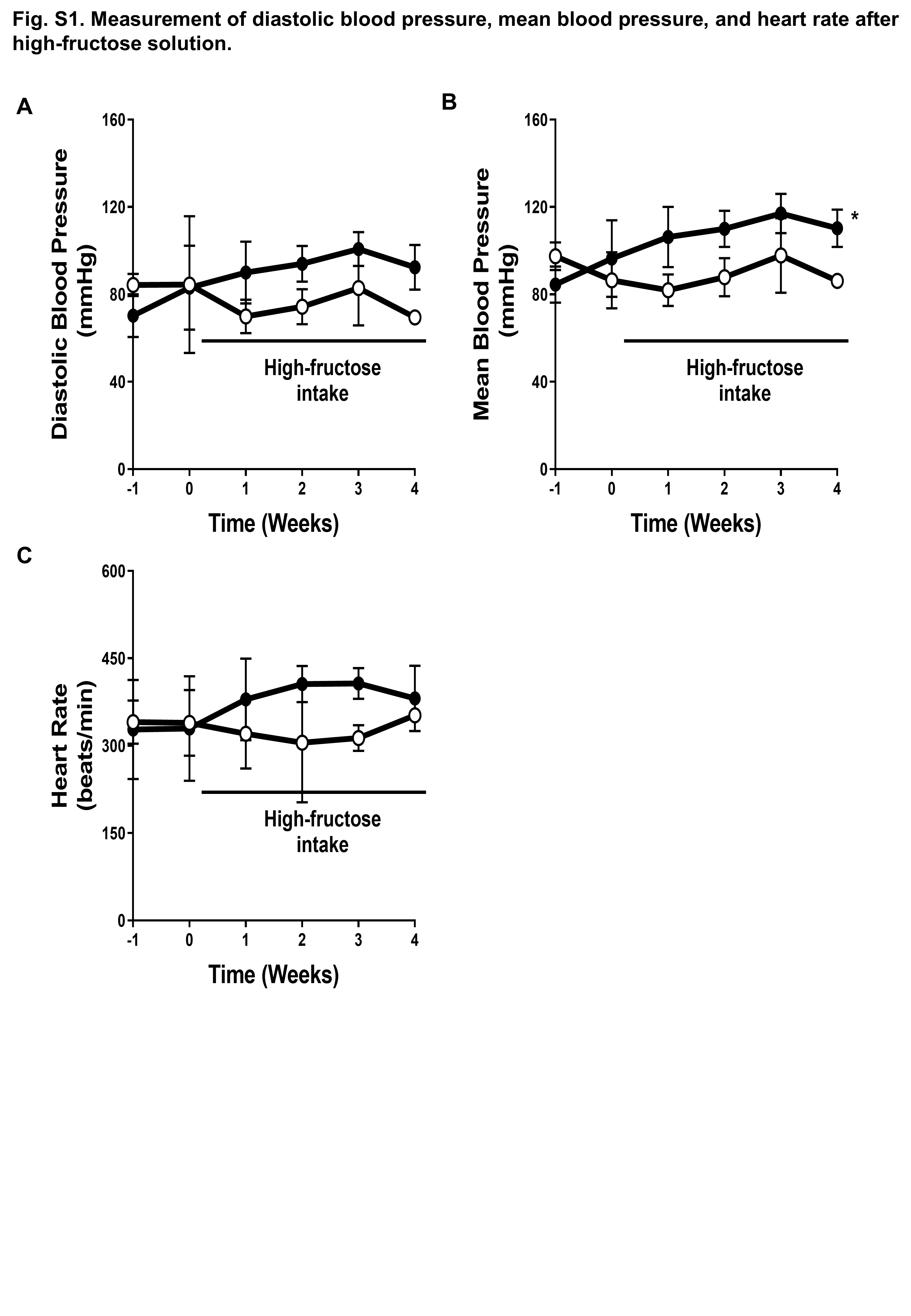


**Supplementary Figure S1. Measurement of diastolic blood pressure, mean blood pressure, and heart rate after high-fructose solution.**

Dahl salt-sensitive (SS) rats were given either 20% high-fructose solution (HFS) or tap water (TW) for 4 weeks. (A) There were no significant differences observed in diastolic blood pressure. (B) The HFS significantly increased mean blood pressure. (C) There were no significant differences observed in heart rate. **Supplementary Figure S2. Calrorie calculation.**


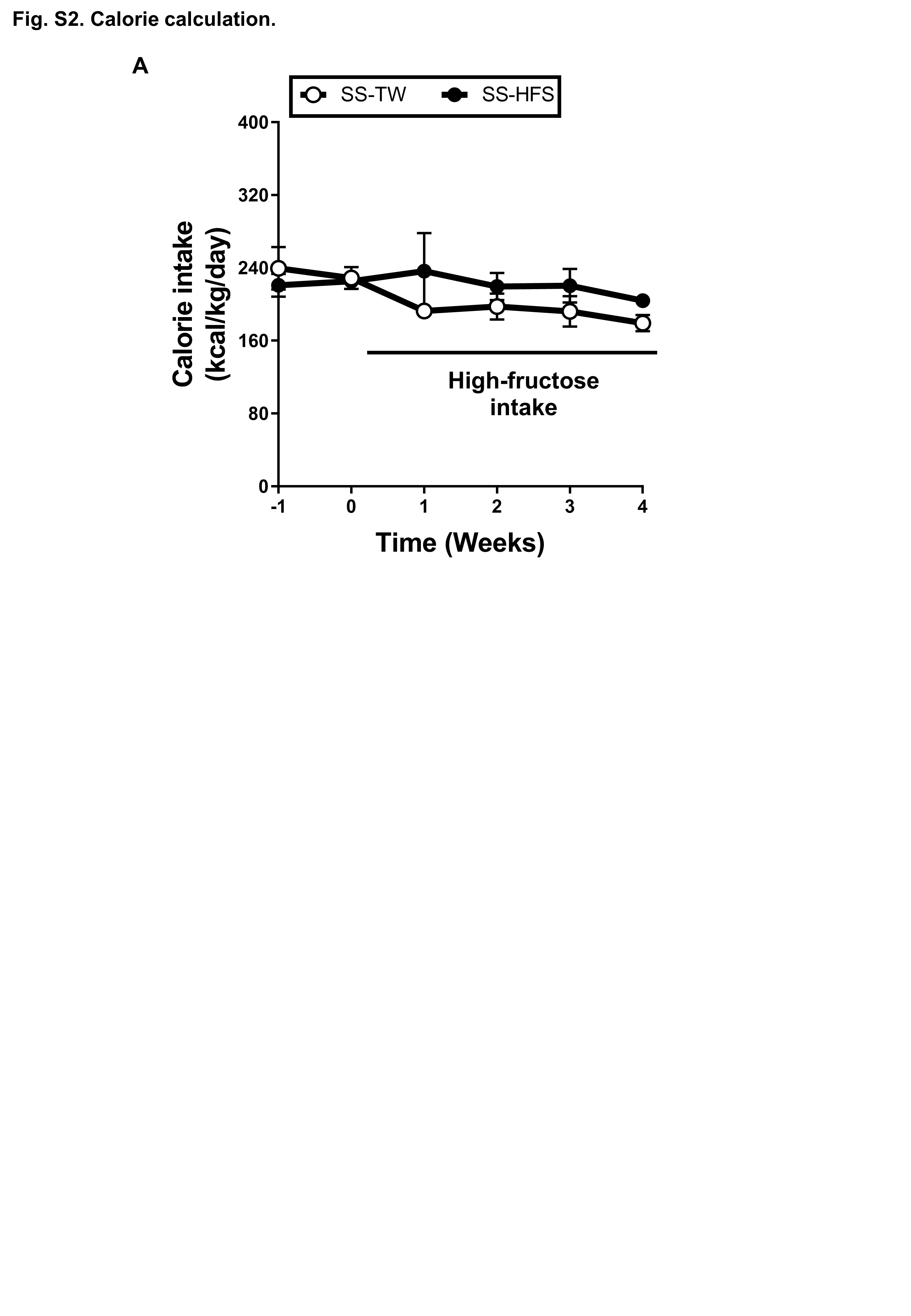


Dahl salt-sensitive (SS) rats were given either 20% high-fructose solution (HFS) or tap water (TW) for 4 weeks. (A) There were no significant differences observed in calorie intake.


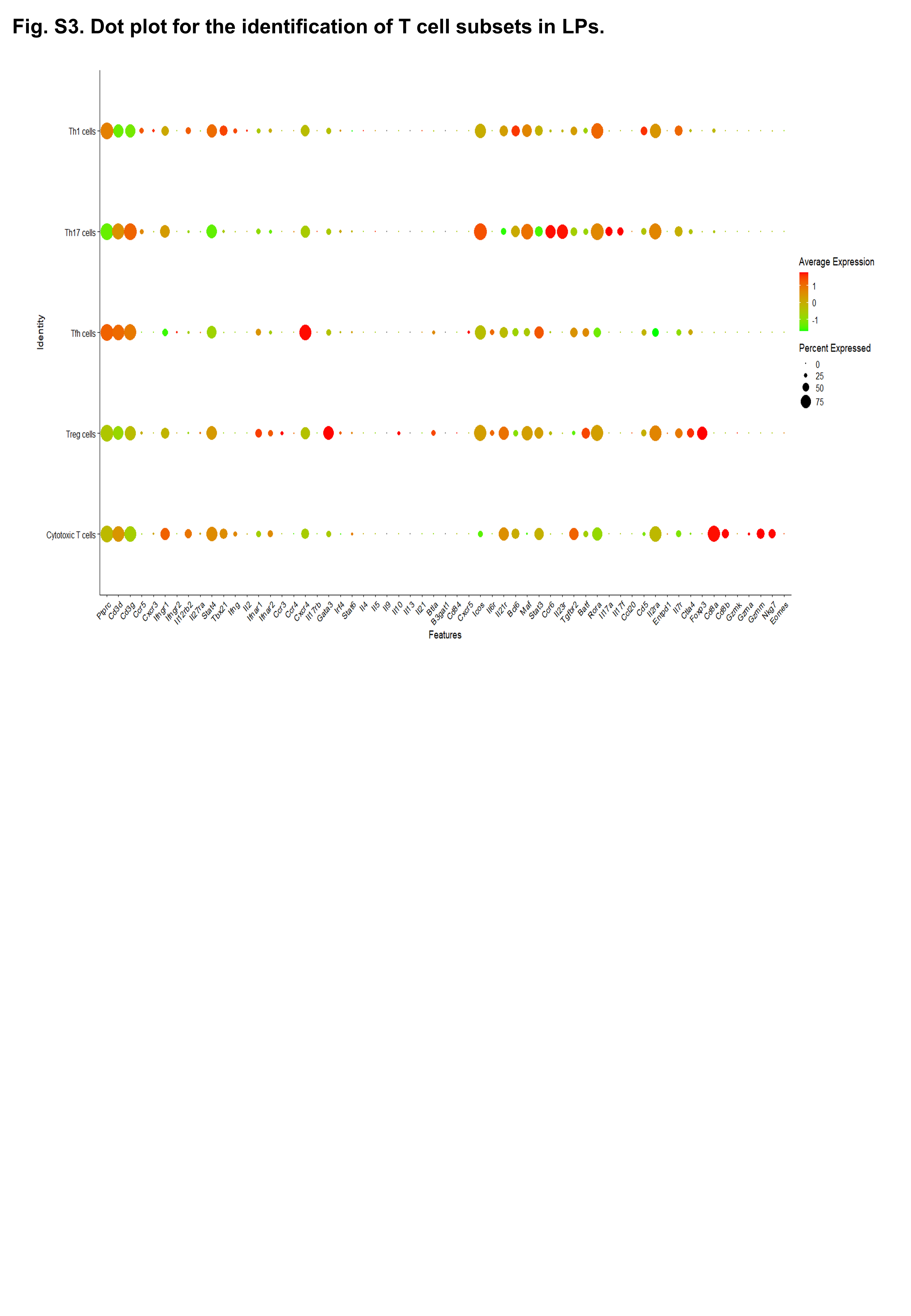


**Supplementary Figure S3. Dot plot for the identification of T cell subsets in LPs.**

The dot plot displays the selected marker genes for each T cell subset in LPs. The size of the dots represents the percentage of gene expression in each T cell subset, while the color of the dots indicates the expression levels.


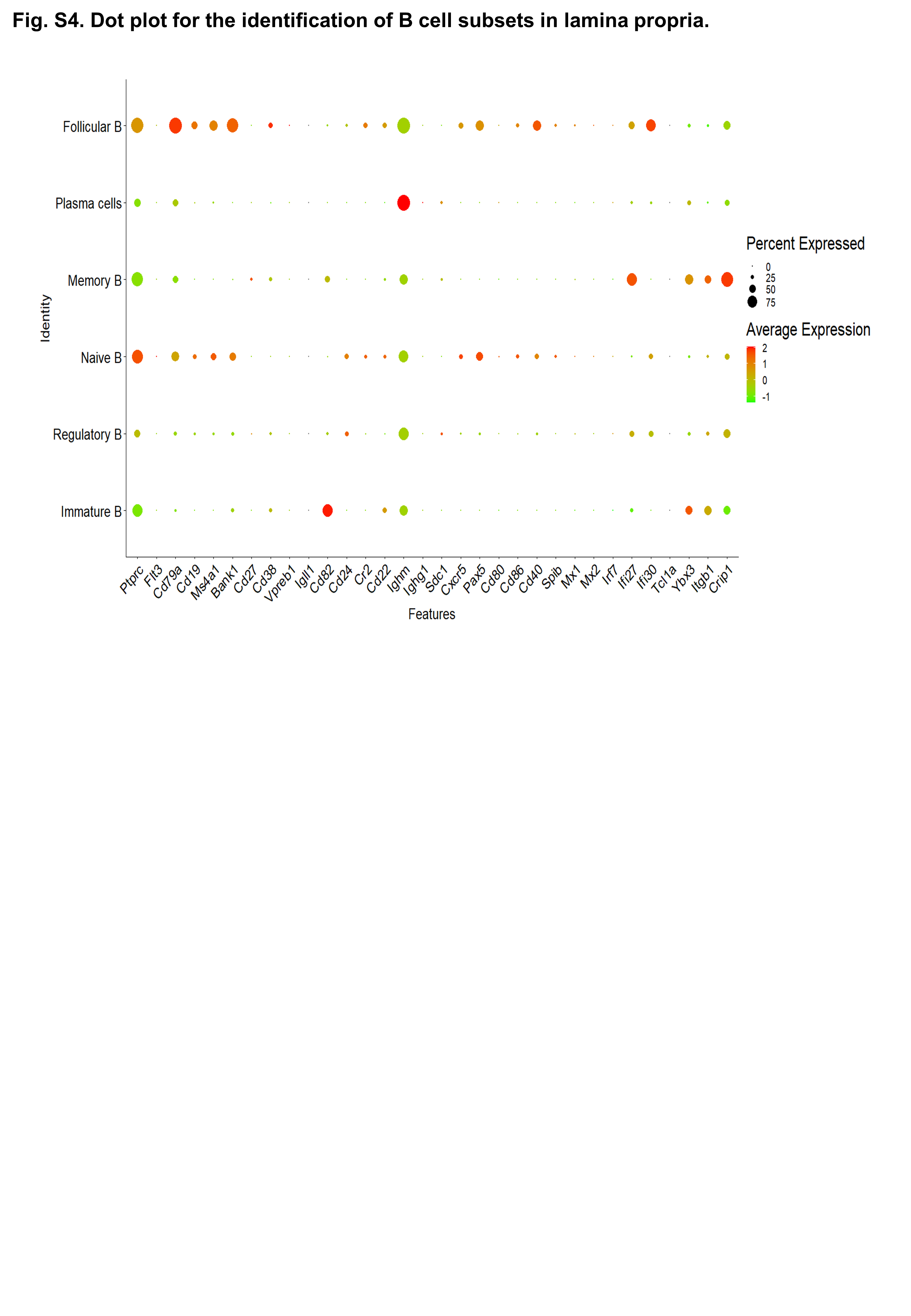


**Supplementary Figure S4. Dot plot for the identification of B cell subsets in LPs.**

The dot plot shows the selected marker genes for each B cell subset in LPs. The size of the dots represents the percentage of gene expression in each B cell subset, while the color of the dots indicates the expression levels.


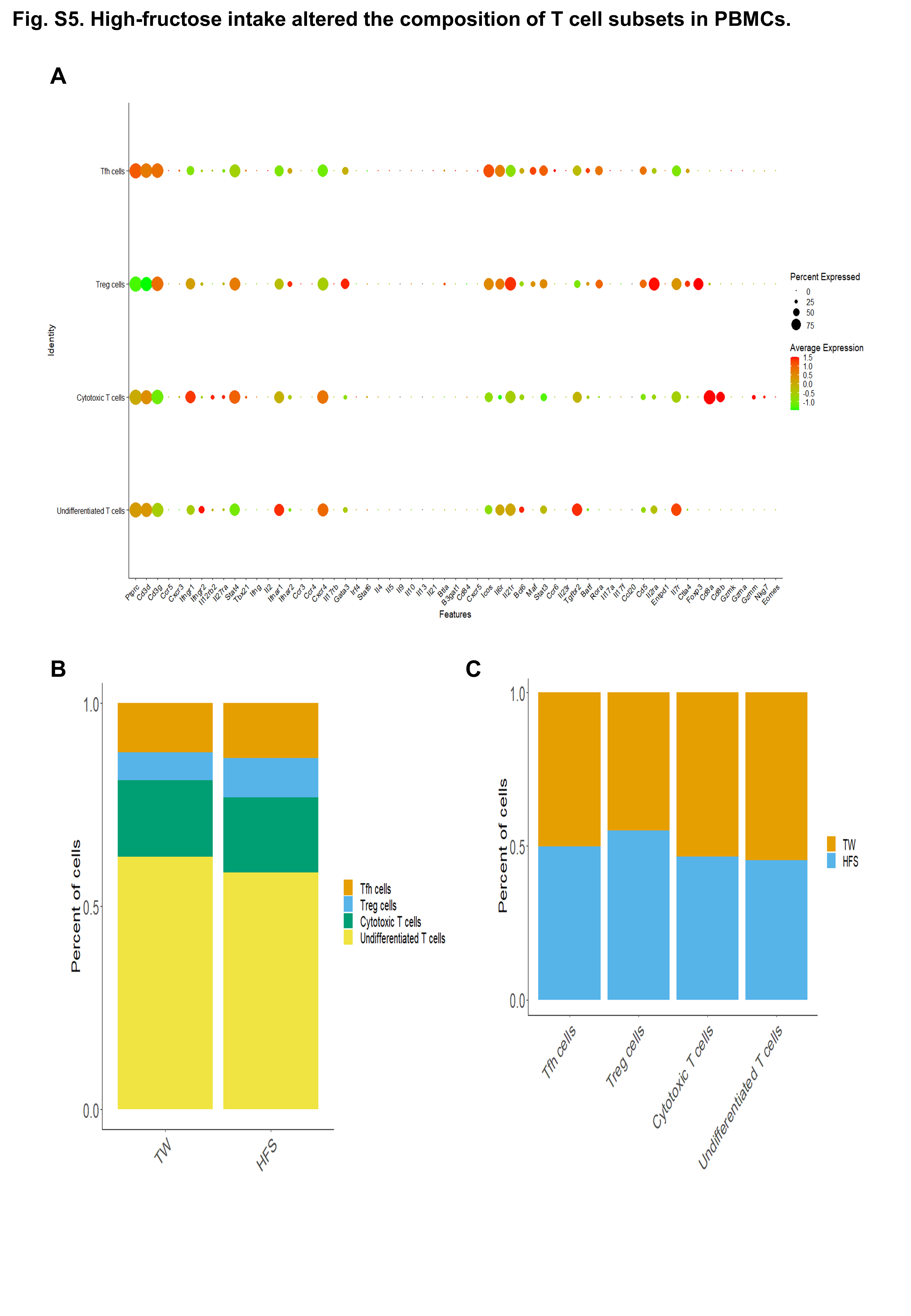


**Supplementary Figure S5. High-fructose intake altered the composition of T cell subsets in PBMCs.**

(A) The dot plot displays the selected marker genes for each T cell subset in PBMCs. The size of the dots represents the percentage of gene expression in each T cell subset, while the color of the dots indicates the expression levels. (B–C) The HFS group exhibited an increase in Treg cells in the T cell subsets of PBMCs compared to the TW group. (B) The proportion of T cell subsets in each group. (C) The relative amount of each T cell subset within the groups.


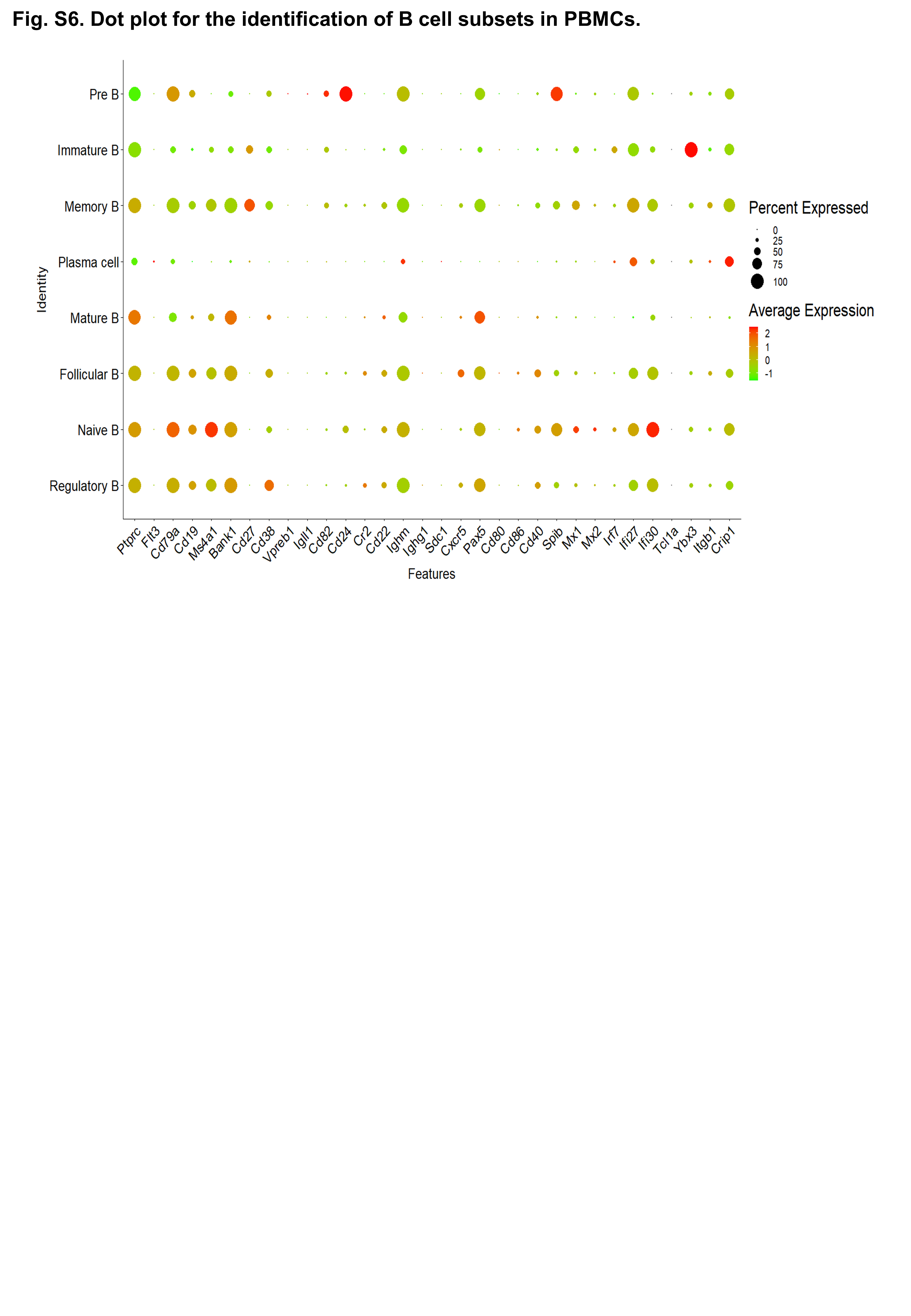


**Supplementary Figure S6. Dot plot for the identification of B cell subsets in PBMCs.**

The dot plot displays the selected marker genes for each B cell subset in PBMCs. The size of the dots represents the percentage of gene expression in each B cell subset, while the color of the dots indicates the expression levels.


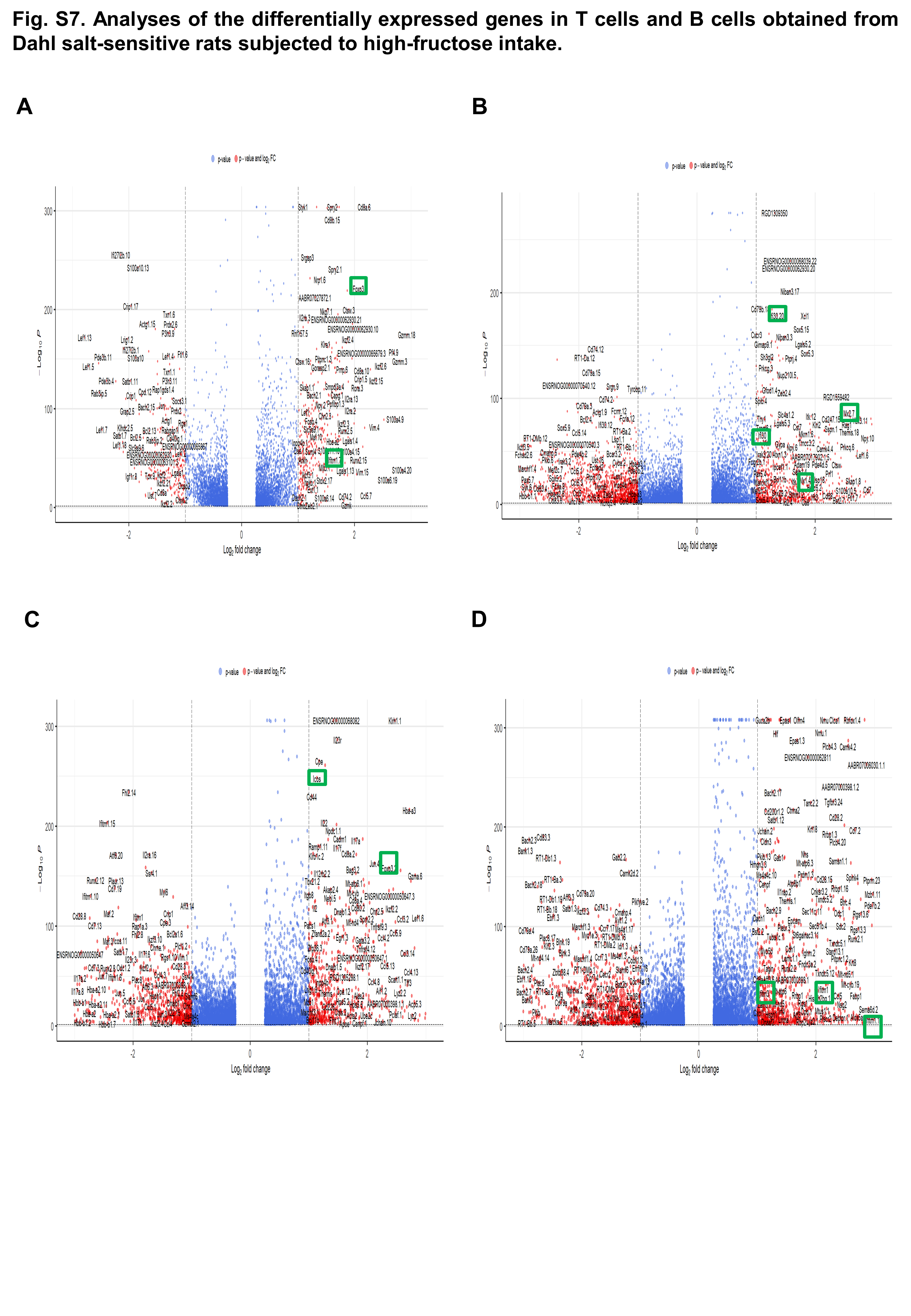


**Supplementary Figure S7. Analyses of the differentially expressed genes in T cells and B cells obtained from Dahl salt-sensitive rats subjected to high-fructose intake.**

(A–B) The enhanced volcano plot displays the log_2_ fold change and -log_10_ *p-value* for each gene in the T cell or B cell clusters of PBMCs. Genes with a significant difference in expression (*p < 0.05*) are represented as red dots. (C–D) The enhanced volcano plot displays the log_2_ fold change and -log_10_ *p-value* for each gene in the T cell or B cell clusters of LPs. Genes with a significant difference in expression (*p < 0.05*) are represented as red dots.

**Supplementary Figure S8. High-fructose intake upregulated the expression of interferon (IFN)-related genes in T cells of PBMCs.**


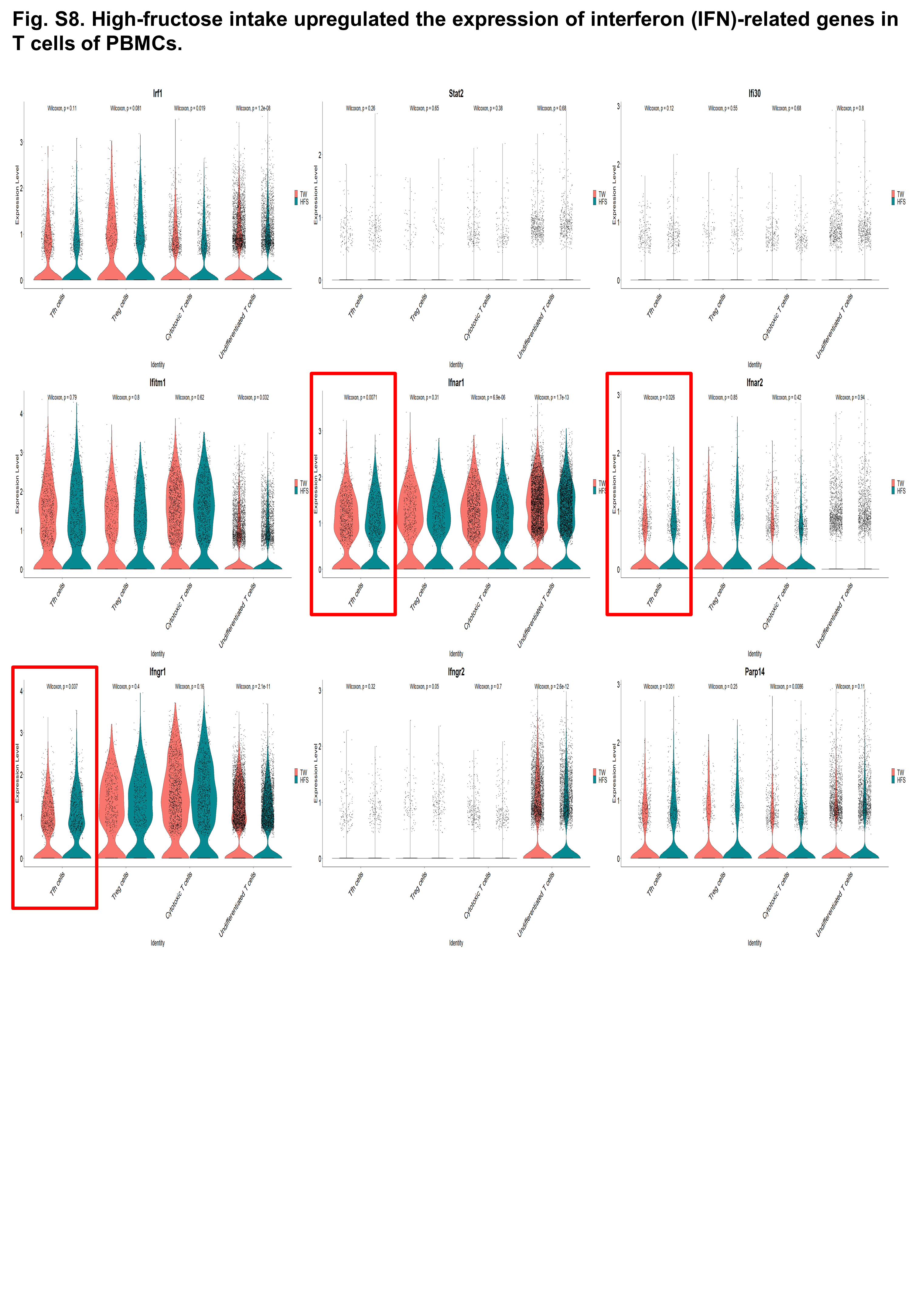


The violin plots display the expression of IFN-related genes such as interferon regulatory factor 1 (*Irf1*), signal transducer and activator of transcription 2 (*Stat2*), interferon-gamma inducible protein 30 (*Ifi30*), interferon-induced transmembrane protein-1 (*Ifitm1*), interferon alpha/beta receptor-1 (*Ifnar1*), interferon alpha/beta receptor-2 (*Ifnar2*), interferon gamma receptor-1 (*Ifngr1*), interferon gamma receptor-2 (*Ifngr2*), and poly (ADP-ribose) polymerase family member 14 (*Parp14*) in each group of T cell subsets from PBMCs.


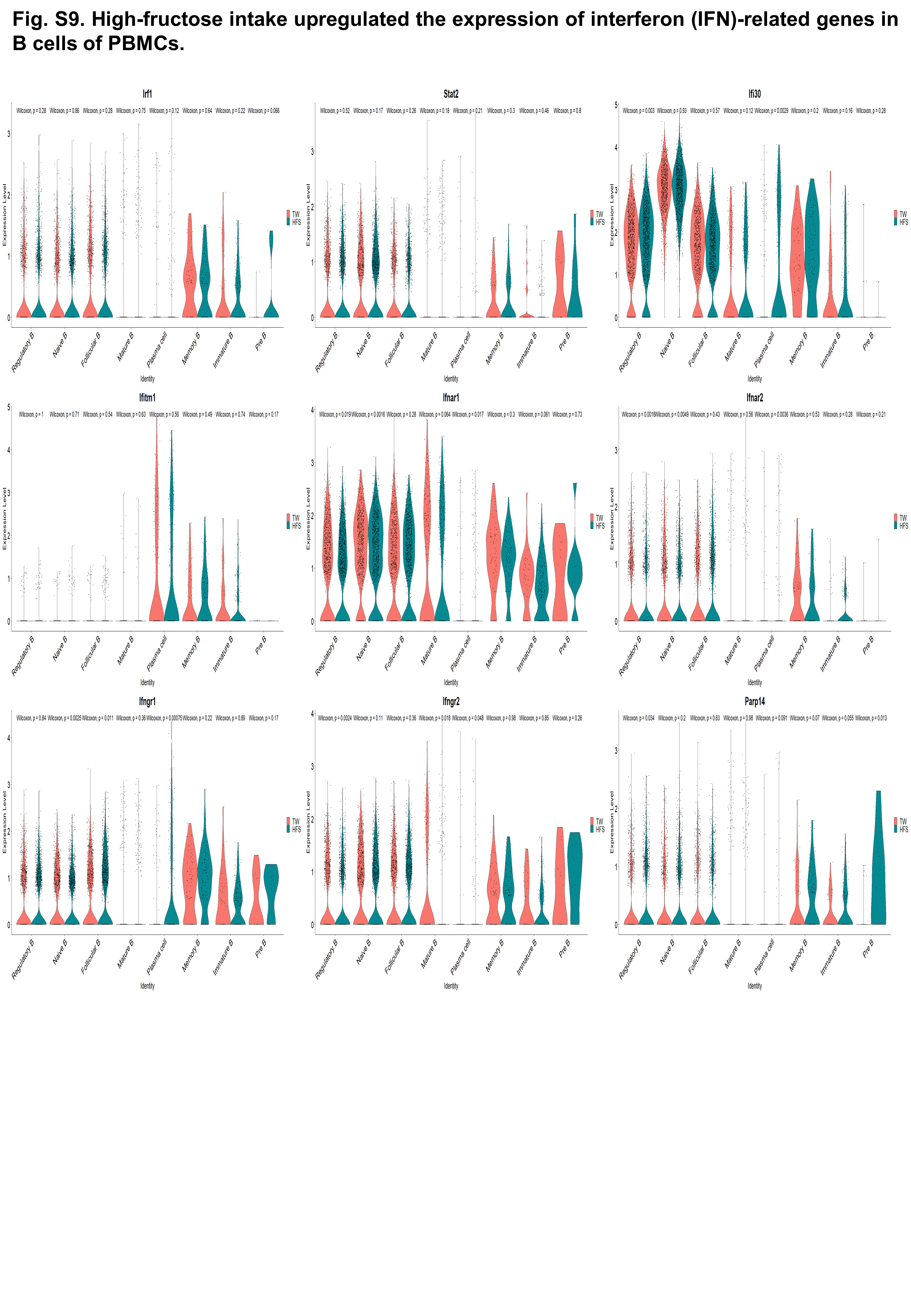


**Supplementary Figure S9. High-fructose intake upregulated the expression of interferon-related genes in B cells of PBMCs.**

The violin plots displays the expression of IFN-related genes such as *Irf1*, *Stat2*, *Ifi30*, *Ifitm1*, *Ifnar1*, *Ifnar2*, *Ifngr1*, *Ifngr2*, and *Parp14* in each group of B cell subsets from PBMCs.


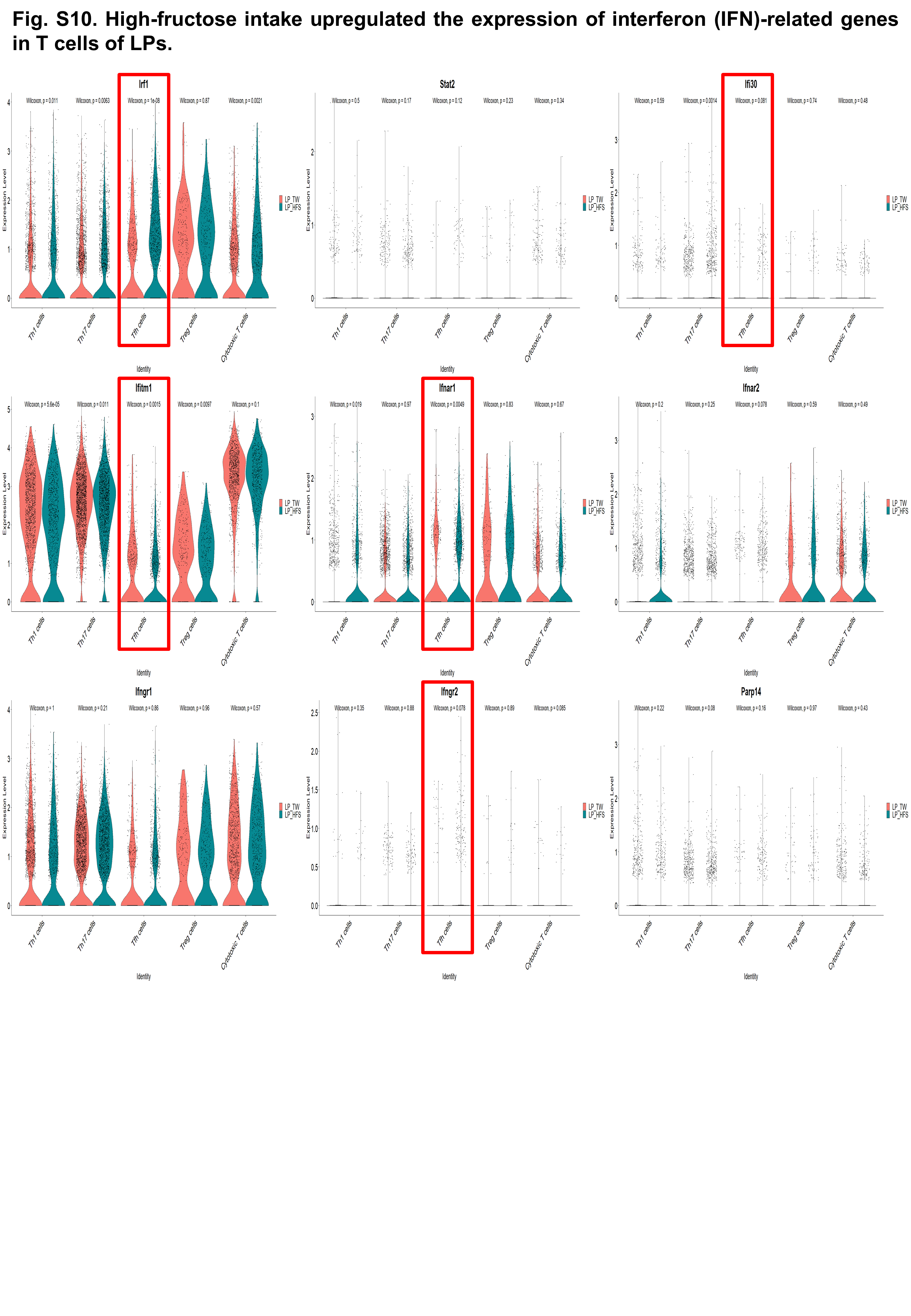


**Supplementary Figure S10. High-fructose intake upregulated the expression of interferon-related genes in T cells of LPs.**

The violin plots display the expression of IFN-related genes such as *Irf1*, *Stat2*, *Ifi30*, *Ifitm1*, *Ifnar1,* *Ifnar2*, *Ifngr1*, *Ifngr2,* and *Parp14* in each group of T cell subsets from LPs.


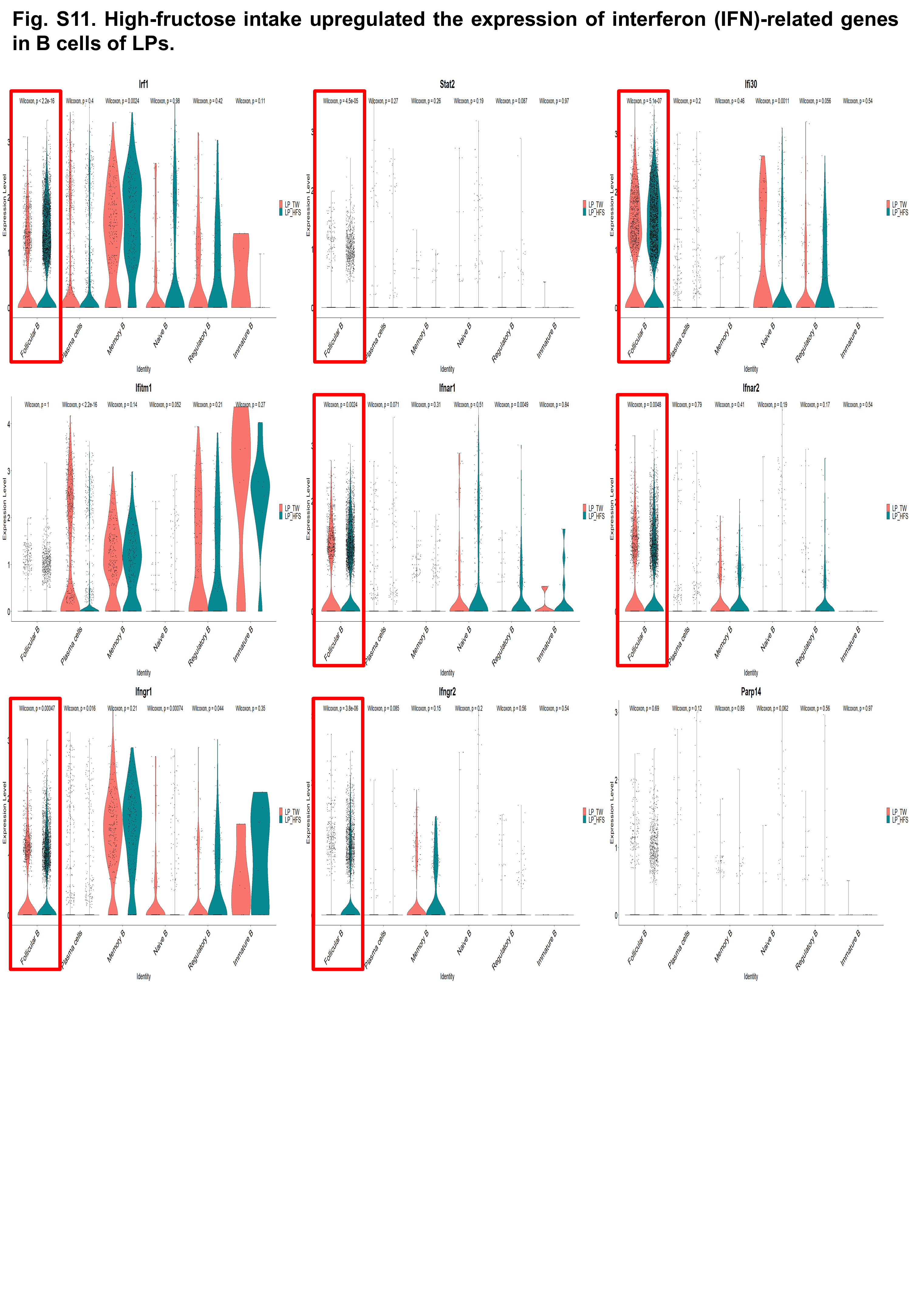


**Supplementary Figure S11. High-fructose intake upregulated the expression of interferon-related genes in B cells of LPs.**

The violin plots display the expression of IFN-related genes such as *Irf1*, *Stat2*, *Ifi30*, *Ifitm1*, *Ifnar1,* *Ifnar2*, *Ifngr1*, *Ifngr2,* and *Parp14* in each group of B cell subsets from LPs.


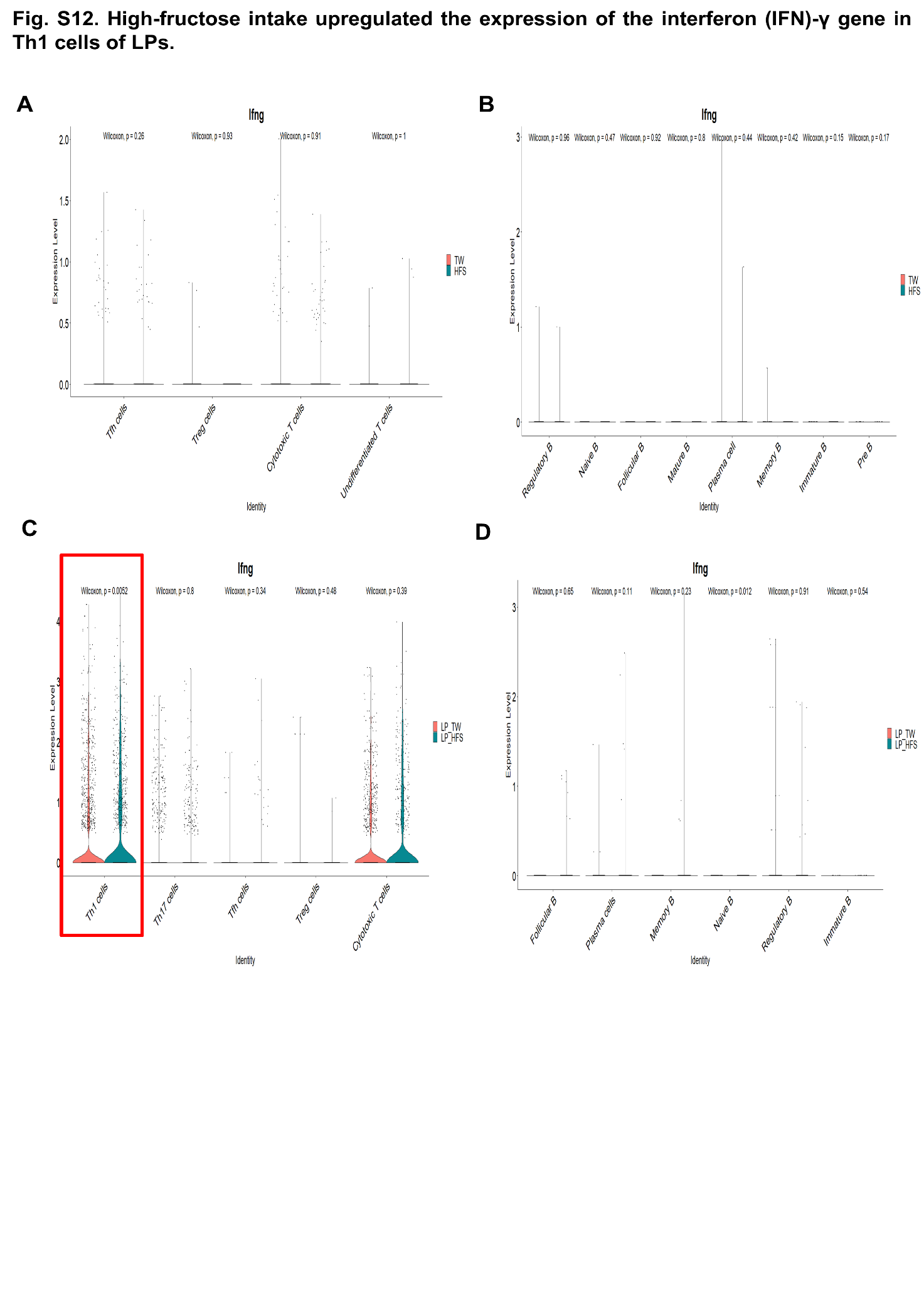
**Supplementary Figure S12. High-fructose intake upregulated the expression of the interferon (IFN)-γ gene in Th1 cells of LPs.**

(A–B) The violin plots display the expression of IFN-γ gene in each group of T cell and B cell subsets from PBMCs. (C–D) The violin plots display the expression of IFN-γ gene in each group of T cell and B cell subsets from LPs.
